# Detection of early-stage NASH using non-invasive hyperpolarized ^13^C metabolic imaging

**DOI:** 10.1038/s41598-024-65951-z

**Published:** 2024-06-27

**Authors:** Cornelius von Morze, Tyler Blazey, Ashley Shaw, William M. Spees, Kooresh I. Shoghi, Michael A. Ohliger

**Affiliations:** 1 Mallinckrodt Institute of Radiology, Washington University, 4525 Scott Ave Rm 2303, St. Louis, MO 63110 USA; 2 Department of Radiology and Biomedical Imaging, University of California, San Francisco, CA USA

**Keywords:** Magnetic resonance imaging, Non-alcoholic steatohepatitis

## Abstract

Non-alcoholic steatohepatitis (NASH) is characterized from its early stages by a profound remodeling of the liver microenvironment, encompassing changes in the composition and activities of multiple cell types and associated gene expression patterns. Hyperpolarized (HP) ^13^C MRI provides a unique view of the metabolic microenvironment, with potential relevance for early diagnosis of liver disease. Previous studies have detected changes in HP ^13^C pyruvate to lactate conversion, catalyzed by lactate dehydrogenase (LDH), with experimental liver injury. HP $\propto$-ketobutyrate ($\propto$ KB) is a close molecular analog of pyruvate with modified specificity for LDH isoforms, specifically attenuated activity with their LDHA-expressed subunits that dominate liver parenchyma. Building on recent results with pyruvate, we investigated HP $\propto$ KB in methionine-choline deficient (MCD) diet as a model of early-stage NASH. Similarity of results between this new agent and pyruvate (~ 50% drop in cytoplasmic reducing capacity), interpreted together with gene expression data from the model, suggests that changes are mediated through broad effects on intermediary metabolism. Plausible mechanisms are depletion of the lactate pool by upregulation of gluconeogenesis (GNG) and pentose phosphate pathway (PPP) flux, and a possible shift toward increased lactate oxidation. These changes may reflect high levels of oxidative stress and/or shifting macrophage populations in NASH.

## Introduction

About one third of the global population is believed to have non-alcoholic fatty liver disease (NAFLD)^1^, or intrahepatic triglyceride content exceeding the normal level required for cellular housekeeping, in the absence of significant alcohol consumption. While also detectable by increased echogenicity on ultrasound, the gold standard method for quantifying liver fat content is MRI proton density fat fraction (MRI-PDFF), in fact providing the widely accepted definition of NAFLD as > 5% MRI-PDFF^2^. Only a subset of ~ 20% patients with NAFLD progress to non-alcoholic steatohepatitis (NASH)^3^, the dangerous hepatic inflammatory state that is perhaps the most relevant clinical entity from the standpoint of early diagnosis of liver disease. NASH carries high risks of transition to critical liver disease states of cirrhosis and/or hepatocellular carcinoma (HCC)^4^, and will soon become the leading cause of liver transplantation^5^. Emerging therapeutic options including pharmaceutical drugs^6^ and bariatric surgery^7^ show significant potential to reverse the course of NASH, if detected early.

Unfortunately, current clinical methods for diagnosis of NASH are extremely limited^8^. The gold standard method for diagnosing NASH is liver biopsy, which is an expensive surgical procedure that carries non-negligible risks to the patient and is therefore unsuitable for screening or tracking this chronic disease over time. Thus there is a pressing need for simpler tests that could serve as early NASH markers. Emerging imaging-based approaches include ultrasound- or MR-elastography^9,10^, and collagen-targeted molecular imaging probes^11,12^. However, these methods primarily target fibrosis, which arises in bulk relatively late in the course of disease. Therapeutic options are more likely to be successful if disease is detected early.

NASH is characterized from earlier disease stages by a profound, dynamic remodeling of the liver microenvironment, including changes in the composition and activities of hepatocyte, stellate, endothelial, and immune cell types^13^. Central molecular features include changes in the expression of genes associated with oxidative stress, immune response, and fibrosis^14^. Large scale displacement of the abundant Kupffer cell population by monocyte-derived macrophages (MoMF’s) has also recently been recognized as an essential feature of NASH^15^, driving its altered bulk transcriptomic profile^16^. The metabolic programming and resulting functions of these MoMF’s is an area of intense current research^17^.

Hyperpolarized (HP) ^13^C MRI is an emerging medical imaging modality for mapping real-time metabolic activity ^18^, especially the proximal metabolic fates of [1-^13^C]pyruvate. The technology is readily translatable into human studies^19^, with an increasing number of research centers having access to the technology. The HP ^13^C scan can be executed as a ~ 2 min add-on to a conventional MRI imaging exam, and does not involve any ionizing radiation. Diffuse liver disease appears to be an especially suitable disease target, as current molecular imaging tools play only a limited current role in this context, with many existing PET tracers displaying non-specific label accumulation in liver. Several preclinical studies have reported large changes in the apparent metabolic conversion of HP [1-^13^C]pyruvate to [1-^13^C]lactate, catalyzed by lactate dehydrogenase (LDH), with liver injury^20,21^. A recent study found that hepatic pyruvate to lactate conversion was decreased in NAFLD/NASH, starting at early disease stages^22^, contrasting with other studies showing lactate *elevation* in hepatotoxic models^20,21^, an alternate dietary model of NAFLD^23^, and diabetes^24^. Although monocarboxylate transporter (MCT) expression is believed to be a major factor in the context of HP tumor imaging^25,26^, it is not clear whether this generalizes to non-malignant tissues. Furthermore, the extent to which hepatic HP signals reflect the metabolic activities of different liver cell types, which are fundamentally reorganized in NASH, is currently unclear. Thus, molecular signatures of early-stage NASH that may be accessible via HP ^13^C MRI require further clarification.

HP [1-^13^C]$\propto$-ketobutyrate ($\propto$ KB) is a close molecular analog of [1-^13^C]pyruvate that exhibits isoform-level selectivity for LDH^27,28^, but otherwise shares many characteristics with pyruvate including high polarization, long T_1_ relaxation time, rapid metabolic rate, and low toxicity. As compared with pyruvate, $\propto$ KB has sharply attenuated activity with “muscle-type” (M) subunits of LDH encoded by LDHA, which are well known to be the dominant form of LDH expressed by liver parenchyma^29^. In comparison, reactivity of $\propto$ KB with “heart-type” (H) subunits of LDH encoded by LDHB remains intact. The differential expression pattern of LDHA vs LDHB throughout the body is commonly rationalized in terms of the differing kinetic characteristics of H-type subunits of LDH (LDHB-expressed), which favor oxidation of lactate, as compared to M-type subunits (LDHA-expressed), which favor lactate production. In this study, we show how the “LDHB-weighting” inherent to HP $\propto$ KB MRI, interpreted together with gene expression data, can inform our understanding of the results of HP liver MRI in the context of early NASH diagnosis.

## Results

### Hyperpolarized ^13^C MRI shows diminished cytoplasmic reducing capacity in early-stage NASH

**Figure 1:**
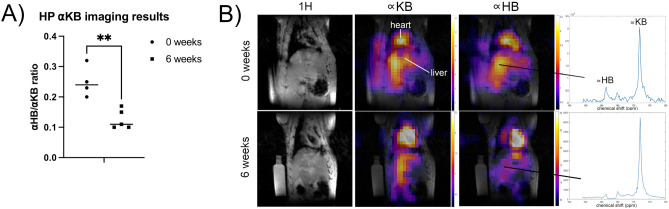
HP [1-^13^C]$\propto$ KB MRI in rodent model of early-stage NASH. (**A**) Summary results showing a mean drop of 52% in the apparent metabolic conversion of $\propto$ KB to $\propto$ HB in liver after six weeks MCD diet (*p* = 0.003). (**B**) Example coronal HP ^13^C metabolite images through rat abdomen, shown as color overlays on ^1^H images in grayscale for anatomic reference. Reference ^1^H images without overlay are shown in leftmost column. Vial containing enriched urea sample for ^13^C signal reference is also seen in these images. Hepatic ^13^C MR spectra from locations indicated by black lines are shown in the rightmost column. The peak at ~ 180 ppm corresponds to a hydrate of $\propto$ KB, not a metabolite.

Representative HP ^13^C metabolite images with corresponding liver-localized spectra and summary data are shown in Fig. 1. The HP $\propto$ HB/$\propto$ KB area under the curve (AUC) ratio in liver was 0.25 $\pm$ 0.05 (mean $\pm$ s.d.) at baseline and 0.13 $\pm$ 0.03 after six weeks MCD diet, meaning that the apparent metabolic conversion of $\propto$ KB to $\propto$ HB was 52% lower in rats with experimental liver injury (p = 0.003, unpaired two-tailed t-test) as compared with baseline. The magnitude of the observed decrement in cytoplasmic reducing capacity is very similar to the prior result with the closely related probe HP [1-^13^C]pyruvate^22^, which reported an analogous decrease in pyruvate-to-lactate conversion of 45% at the same six week time point.

### MCD diet induces early-stage NASH in rats within six week

**Figure 2:**
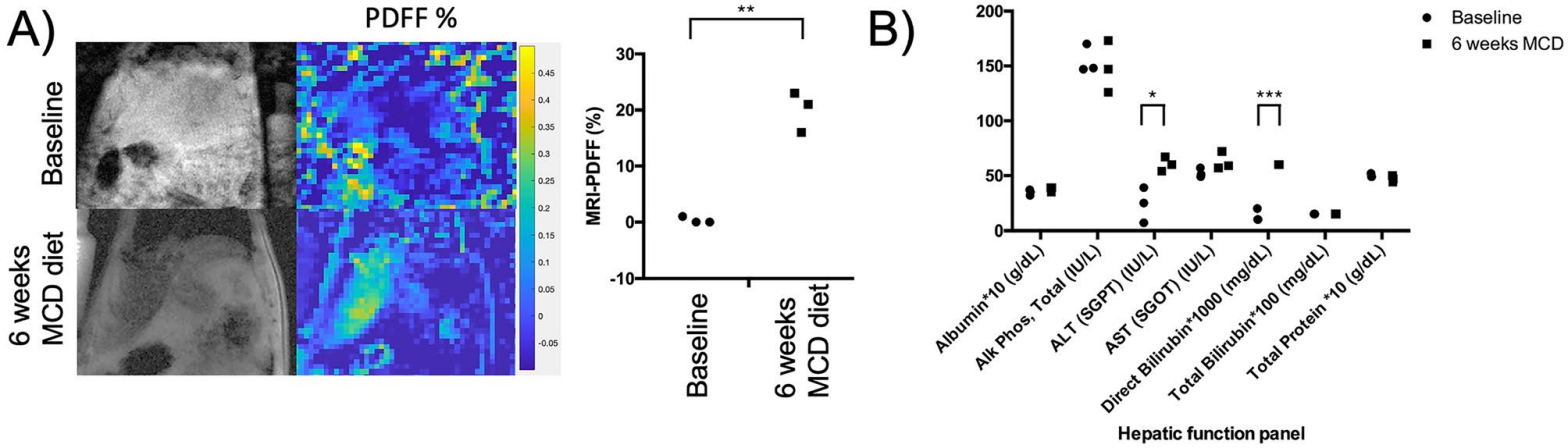
Conventional non-invasive clinical markers of NAFLD/NASH in rodent model of early-stage NASH. (**A**) Example ^1^H MRI-PDFF images and group data showing elevation of hepatic fat signal fraction in rats fed MCD diet (0.33 $\pm$ 0.57 to 20.0 $\pm$ 3.6%, p = 0.001). (**B**) Results of hepatic function panel showing elevated liver enzymes in rats fed MCD diet (ALT: 24 $\pm$ 16 to 60 $\pm$ 6.5 IU/L, p = 0.02; AST: 52 $\pm$ 4.2 to 63 $\pm$ 8.1 IU/L, p = 0.12).

**Figure 3:**
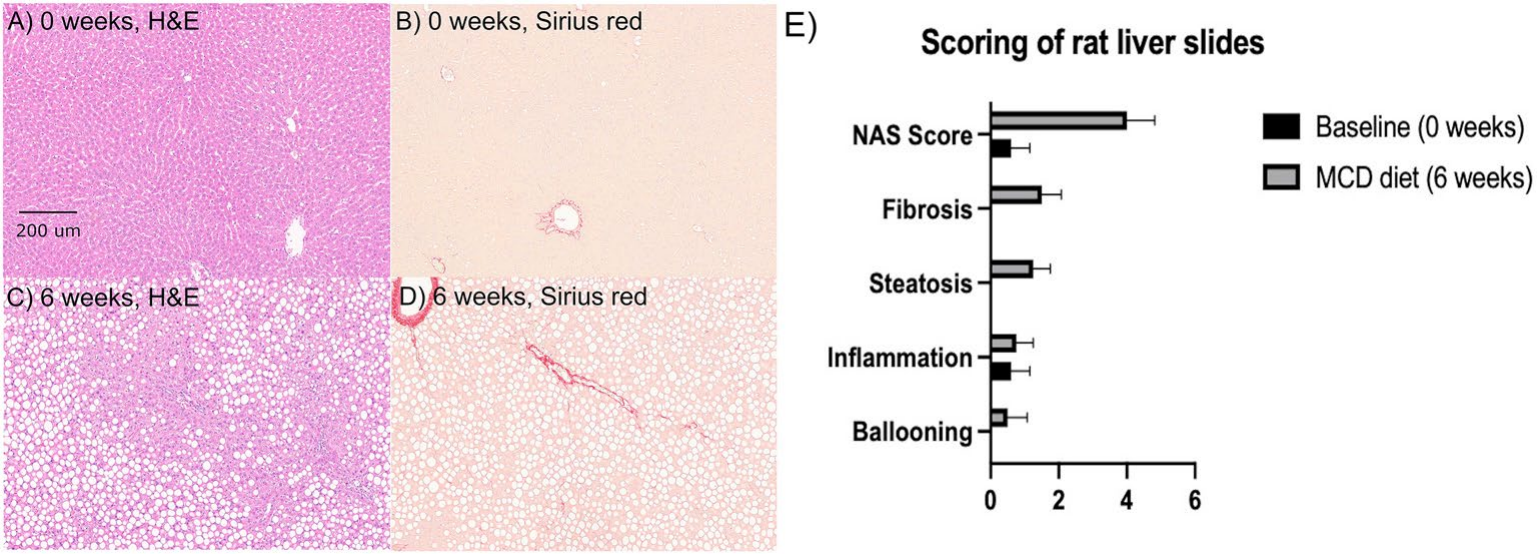
Histopathology of NAFLD/NASH in rodent model of early-stage NASH. (**A**) Example stained liver tissue sections demonstrating histologic features of NAFLD/NASH in livers harvested from MCD model. H&E (**A**, **C**) and Sirius red (**B**, **D**) stained sections are shown. Specific observations are extensive macrovesicular steatosis, sparse inflammatory foci, and moderate excess collagen deposition (Sirius red). (**E**) Automated discrete scoring of Trichrome slides demonstrates elevated NAS scores in rodents fed MCD diet, as well as increased fibrosis (NAS: 0.60 $\pm$ 0.55 to 4.0 $\pm$ 0.8, p = 0.0001). Error bars show s.d.’s of the sample means.

Consistent with numerous prior studies^22,30,31^, MCD diet was effective at inducing severe NAFLD in rats within a short period of time. As depicted in Fig. 2A, MRI-PDFF showed a large increase in triglyceride ^1^H MRI signals in rat liver from 0.33 $\pm$ 0.57 to 20.0 $\pm$ 3.6% (p = 0.001) at six weeks. The appearance of hepatic steatosis was accompanied by a mean weight loss of 15 $\pm$ 0.7% over the six weeks (p = 0.003), a known effect of the MCD model. As depicted in Fig. 2B, venous plasma sampling revealed elevation of liver enzymes consistent with liver injury. Specifically, ALT increased from 24 $\pm$ 16 to 60 $\pm$ 6.5 IU/L (p = 0.02), and AST trended upward from 52 $\pm$ 4.2 to 63 $\pm$ 8.1 IU/L (p = 0.12). Histology also revealed clear evidence of fatty liver disease (Fig. 3). Most evident was extensive macrovesicular steatosis at six weeks on diet, seen as the white droplets in Figs. 3C, D. Modest scarring was also detectable, as were sparse inflammatory foci. Computerized pathology readings, summarized in Fig. 3E, indicated early NASH. NAS scores were significantly elevated from 0.60 $\pm$ 0.55 at 0 weeks to 4.0 $\pm$ 0.8 at 6 weeks (p = 0.0001). Note that the accepted cutoff for human NASH is a NAS score of 5^32^.

### Early NASH transcriptome reveals potential mechanisms of diminished cytoplasmic reducing capacity

**Figure 4:**
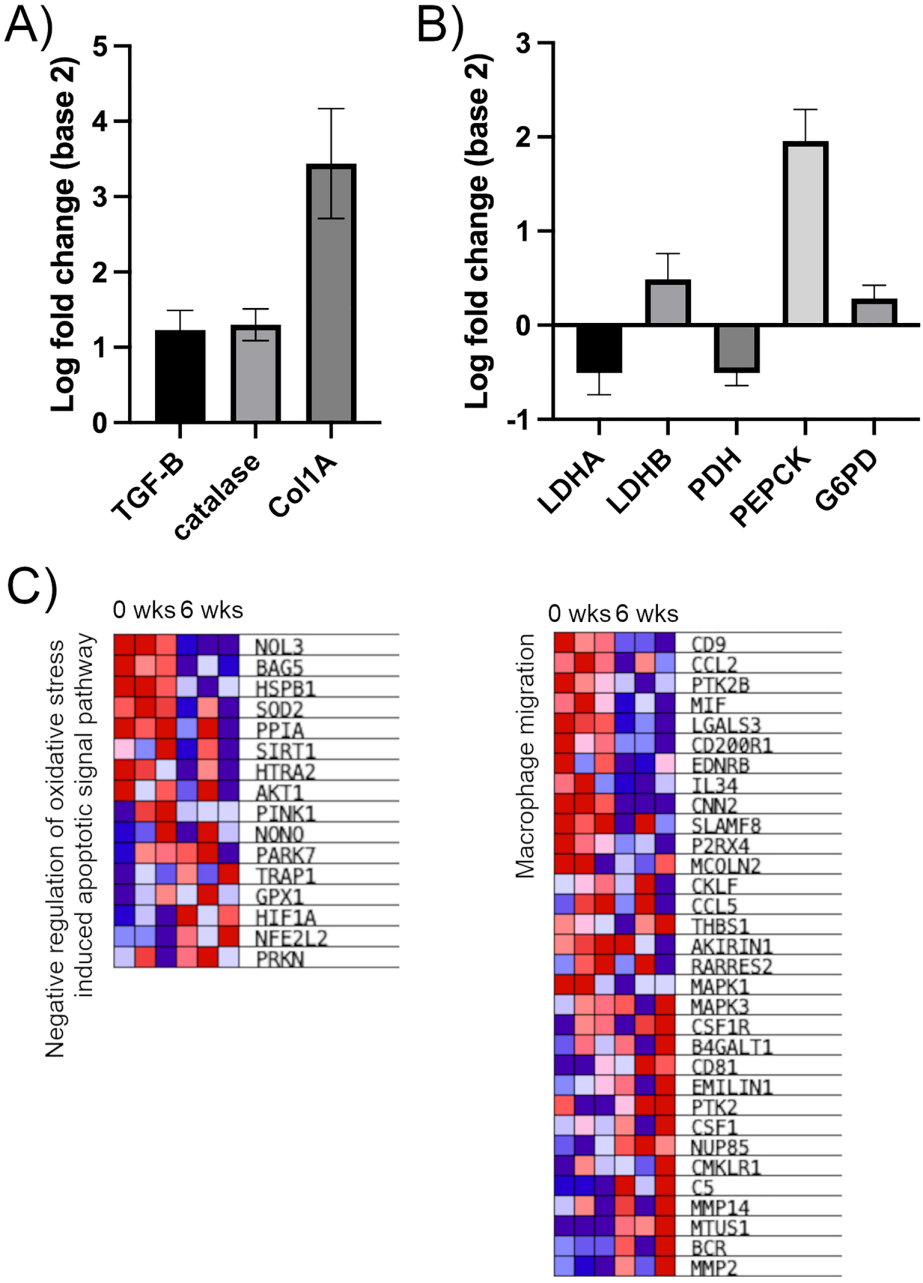
Changes in liver gene expression in rodent model of early-stage NASH. (**A**) Upregulation of selected genes associated with inflammation (transforming growth factor or TGF-$\beta$), oxidative stress (catalase), and fibrosis (alpha-1 type-1 collagen or Col1A) in MCD model. (**B**) Changes in gene expression proximal to pyruvate metabolism with relevance to HP MRI results. (**C**) Heat maps for two highly enriched gene sets, related to oxidative stress-induced apoptosis (left) and macrophage migration (right).

Bulk RNAseq detected significantly different expression of 6949/10,947 genes in rats with early-stage NASH. We first checked hallmark genes associated with oxidative stress and fibrosis, to confirm expected changes associated with liver injury (Fig. 4A). We next examined differential expression of individual genes proximal to pyruvate metabolism (Fig. 4B), to look for factors that could potentially provide a direct explanation for the HP results. Expression of MCT1&4 was not significantly different between groups. Notably, LDHB was upregulated while LDHA was downregulated, which could indicate a shift toward increased lactate oxidation in early-stage NASH. Of largest quantitative significance among these genes, the key cataplerotic enzyme PEPCK (rate-limiting step of gluconeogenesis or GNG) was upregulated 3.9-fold, which would tend to deplete TCA intermediates and consequently also the lactate pool, potentially decreasing the apparent metabolic conversion of $\propto$ KB and pyruvate by depleting the pool of lactate available for isotopic exchange flux^33,34^. We also detected a modest upregulation of G6PD (rate-limiting step of pentose phosphate pathway or PPP) that could also tend to draw carbon away the lactate-pyruvate pool.

Gene set analysis contextualizes these results with lower dependence on changes in any particular gene or metabolic segment. Of 2222 total gene sets analyzed, among the top seven most highly enriched GOBP gene sets in rats with early-stage NASH were “Negative regulation of oxidative stress induced intrinsic apoptotic signaling pathway” (NES = 2.06, p < 0.001, FDR q-val = 0.067) and “Macrophage migration (NES = 2.00, p < 0.001, FDR q-val = 0.10). These enrichments (Fig. 4C) highlight the established role of excess ROS production^35,36^, as well as recent findings about shifting inflammatory cell populations in NASH pathogenesis^15^.

## Discussion

This study showed that HP ^13^C $\propto$ KB can be used to detect hepatic metabolic changes in early NASH. LDHB-weighted reduction of $\propto$ KB to $\propto$ HB was attenuated to a very similar extent as the reduction of pyruvate to lactate in a recent study that employed the same model of early NASH^22^, despite changes in the relative expression levels of LDHA and LDHB. This suggests a limited direct role for LDH expression in HP contrast in the present context. More likely, the apparent fluxes of either probe along the accessible proximal segments are influenced by wider effects on intermediary metabolism.

Evidence from our gene expression data suggests that increased GNG and PPP flux in early NASH could each deplete the lactate pool in a manner that could lead to the observed result, by limiting isotopic exchange flux^33,34^. Increased expression of LDHB relative to LDHA in early NASH could also contribute to such an effect by favoring lactate oxidation. Of course, gene expression is not to be equated with metabolic fluxes, or even protein levels. Besides protein levels, metabolic fluxes can also depend on substrate concentrations, allosteric interactions, enzyme compartmentalization, and posttranslational modification (phosphorylation), among other factors. Nevertheless, many enzymes are known to be regulated at the level of transcription, including PEPCK^37^ and, to a lesser extent, G6PD^38^. Thus, the changes in mRNA levels of these two enzymes probably do actually result in increased GNG and PPP flux. These pathways may be less important in the other models of liver injury that reported lactate elevation^20,21,23,24^.

The observed upregulation of GNG and PPP pathways is likely related to high levels of oxidative stress, which is a key pathway of liver injury in NASH through oxidative stress-induced apoptosis^39^. Elevated mitochondrial anaplerotic/cataplerotic activity is coordinated energetically with oxidative metabolism, coupling elevated GNG to higher levels of oxidative stress^40,41^. Moreover, GNG can be induced directly by oxidative stress^42^. The PPP generates NADPH in part to combat oxidative stress, and is thought to be activated directly in response to oxidative stress via the NADPH/NADP^+^ ratio^38^. PPP flux can also be induced by acute application of oxidative stress^43^. Obese Zucker rats have been shown to have higher hepatic PPP flux than lean controls at 8 weeks, but not at 16 weeks, highlighting the importance of this pathway in early disease^44^. A previous study applied the HP ^13^C probe [1-^13^C]dehydroascorbate to directly probe hepatic oxidative stress in the MCD diet^45^. Interestingly, an analogous drop in the reduction of DHA to vitamin C was observed in rodents with NAFLD/NASH in that study, in a reaction where the alternate co-factor NADPH is the electron donor.

It is important to evaluate our results in the context of multiple quantitatively important cell types present in liver. Hepatocytes constitute the largest liver cell fraction, but Kupffer cells are the next largest and account for ~ 15% of all liver cells, representing the single largest resident macrophage population in the body^46^. Considering the high metabolic capacity of macrophages and their high cell fraction in liver, macrophages could account for some part of the metabolic changes detected by HP imaging. In contrast with hepatocytes, macrophages (both resident Kupffer cells and MoMF’s) are known for highly plastic expression programs shaped by their local microenvironment^47^, and can express LDHA and/or LDHB^48^. The classical M2 polarization state of macrophages favors lactate oxidation and is regarded to be anti-inflammatory^49^, and could be reflected in the opposite changes in expression of LDHA&B detected in early NASH in this study. Thus, replacement of the resident Kupffer cell population with MoMF’s^15^ may also have contributed to the detected metabolic shift.

Six weeks on MCD diet was confirmed as an appropriate time point for modeling early NASH in rats through blood testing and automated pathology readings^50^, in agreement with prior results with the same model^22^. Automated detection of inflammation was less reliable than any of the other scored pathology criteria, likely stemming from the inherent difficulty of detecting inflammatory foci and distinguishing these cells from erythrocytes, together with the limited available training data and potential deviations from our data such as differences in the details of staining techniques between labs.

Although it effectively mimics several aspects of human NASH over a short period of time, the MCD diet has some important limitations. The biggest limitations of the MCD model are the significant weight loss exhibited during disease induction and the absence of insulin resistance, both us which are inconsistent with the typical picture of human NASH, which has tight associations with obesity and type 2 diabetes^51^. Human NASH, representing sustained liver damage over long periods of time, is known to be more severe histopathologically than existing NASH models. It is difficult if not impossible to cause cirrhosis in rodents without repeated toxic insults. Only male rats, which are known to develop greater liver injury on the MCD diet in comparison with females^52^, were included in this study.

HP ^13^C data should be interpreted as only one part of the overall MRI scan session, of which the HP ^13^C scan is an add-on component. The ^1^H-based MRI-PDFF scans in this study showed large signal changes on MCD diet, much larger fractionally than the HP ^13^C changes, with the prior study by Piraquive et al.^22^ showing that MRI-PDFF changes more rapidly than HP ^13^C metabolite ratios. However, depiction of the presence and amount of liver fat has limited value for detection of NASH, with liver biopsy remaining the gold standard. Arguably, the biggest potential added value for molecular imaging methods such as HP ^13^C in this context would be in differentiating benign fatty liver from fatty liver with inflammation (i.e., NASH). This could be assessed for the $\alpha$ KB probe by adding more study time points with matching pathology readings.

In summary, HP ^13^C MRI provides metabolic readouts of early-stage NASH in rodent models, although underlying contrast mechanisms and relevance to human disease have yet to be fully established.

## Methods

### Animal model

A total of 14 adult male Sprague Dawley rats (Charles River) were studied, starting at 12 weeks of age. A sub-group of these rats were imaged at baseline (n = 4) and after six weeks on a custom diet deficient in methionine and choline (n = 5) (MCD diet, Envigo TD.90262), a frequently utilized model of NAFLD/NASH^30,31^. Fixed and frozen liver tissue samples were harvested at both time points in sub-groups of rats (fixed—n = 5 at baseline, n = 4 at 6 weeks; frozen—n = 3 at baseline, n = 3 at 6 weeks). Venous plasma samples were also collected in sub-groups at both time points (n = 3 at baseline, n = 3 at 6 weeks). All experimental protocols were approved by the Institutional Animal Care and Use Committee (IACUC) at Washington University (protocol #22-0054), and all methods were carried out in accordance with relevant guidelines and regulations. All methods are reported in accordance with the ARRIVE guidelines.

### ^13^C/^1^H MRI

MRI scanning was conducted in a compact, cryogen-free PET/MR system (MR Solutions, Guildford, UK) equipped with a dual-tuned ^13^C/^1^H radiofrequency coil designed for imaging rats. This system has a variable magnetic field strength of 7 T/3 T, and was ramped to 3 T for the present study. For each imaging study, 130 $\mu$L [1-^13^C]$\propto$ KB acid mixed with 15 mM trityl radical OX063 was hyperpolarized using a GE 5 T SPINlab (GE Healthcare). At the time of the HP experiment, each HP sample was dissolved with 18 mL superheated water and neutralized using NaOH/Tris buffer. 2.4 mL contrast agent was injected into the rat tail vein over 10 s. HP ^13^C images through rat abdomen were acquired using a previously described flyback echo planar spectroscopic imaging (EPSI) pulse sequence ^53^, with spatial resolution of 5 mm × 5 mm × 20 mm and temporal resolution of 4 s. Multi-band spectral-spatial RF excitation was used to apply a smaller flip angle of 5 $^\circ$ on the primary substrate [1-^13^C]$\propto$ KB and 12$^\circ$ on [1-^13^C]$\propto$ HB, conserving the magnetization of the primary substrate for subsequent metabolic conversion^54^. Metabolite data was quantified by regional integration and summation over time to produce metabolite AUC ratios. To quantify hepatic triglyceride content, multi-echo ^1^H GRE images were acquired with six echoes with TE_1_ = 2.21 ms and $\Delta$TE = 1.68 ms. Resulting data were fit using the LIPOQUANT (Liver Imaging of Phase interference related signal Oscillation and QUANTification) package in MATLAB to compute MRI-PDFF. T_1_-weighted MRI images were also collected for anatomic reference.

### Blood testing

To measure conventional markers of liver injury, venous blood plasma samples were collected for a hepatic function panel conducted by a CLIA certified laboratory. The panel measured albumin, alkaline phosphatase, alanine transaminase (ALT), aspartate aminotransferase (AST), direct and total bilirubin, and total protein.

### Pathology

Formalin fixed, paraffin embedded liver tissue sections were stained with H&E, Sirius red, and Mason’s trichrome, and whole slides were digitized at 20 × magnification. Digital trichrome data were analyzed using a “pathologist-like” deep learning (DL) system that outputs discrete NAFLD-related histological feature scoring based on hepatocyte ballooning (0–2), inflammation (0–2), steatosis (0–3), and fibrosis (0–4)^32,50^. The code analyzes trichrome tiles (299 × 299 px^2^, resampled to 0.44 $\mu$m/px) with convolutional neural networks (CNN’s) trained using gold standard scoring of thousands of tiles by an expert pathologist. All feature detection models were used without modification, except for an adjustment to the inflammation score cutoffs. The NAFLD Activity Score (NAS) was computed as the sum of scores for ballooning, inflammation, and steatosis, in the usual manner utilized by the NASH Clinical Research Network (CRN)^32^.

### RNAseq

 > 30 $\mu$g RNA was isolated from each frozen liver tissue sample. The bulk RNAseq procedure included library preparation based on poly(A) enrichment, ~ 30 M 2 × 150 reads from the NovaSeq S4 system. Gene set enrichment analysis was performed on the resulting mRNA expression data using GSEA (version 4.3.2), with the C5 gene ontology (GO) biological processes (BP) gene set (v2023). Rat genes were mapped to human orthologs based on MSigDB (v2023).

## Data Availability

The datasets generating during and/or analyzed during the current study are available from the corresponding author on reasonable request.
